# Integrated GC–MS and UHPLC–HRMS/MS profiling of bioactive compounds from *Streptomyces* sp. BPA-6 isolated from bees-collected pollen

**DOI:** 10.1007/s11306-026-02427-3

**Published:** 2026-05-24

**Authors:** Hani Belhadj, Mohamed Mokhnache, Ahmed Mohamed Bachir Alien, Marika Pellegrini, Sara Palmieri, Federico Fanti, Daoud Harzallah, Tarek H. Taha, Walid Elfalleh, Stefania Garzoli, Hamdi Bendif

**Affiliations:** 1 Laboratory of Applied Microbiology, Faculty of Natural and Life Sciences, University Ferhat Abbas Setif 1, 19137 Setif, Algeria; 2 Department of Life, Health, and Environmental Sciences, University of L’Aquila, 67100 L’Aquila, Italy; 3 Department of Bioscience and Technology for Food, Agriculture and Environment, University of Teramo, Via Renato Balzarini 1, 64100 Teramo, Italy; 4 Department of Biology, College of Science, Imam Mohammad Ibn Saud Islamic University (IMSIU), 11623 Riyadh, Saudi Arabia; 5 Department of Chemistry and Technologies of Drug, Sapienza University, P. Le Aldo Moro, 5, 00185 Rome, Italy

**Keywords:** Actinomycetes, *Streptomyces*, Antimicrobial resistance, Antimicrobial activity, Secondary metabolites

## Abstract

Antimicrobial resistance is one of the most current global health challenges, due to the increasing ineffectiveness of antibiotics and the increasing difficulty in treating infections. Approximately two-thirds of microbial antibiotics are produced by actinomycetes, of which approximately 74% come from the genus *Streptomyces*. In this study, a novel actinomycete strain, *Streptomyces* sp. BPA-6, isolated from pollen collected by Algerian bees, was investigated for its antimicrobial potential and chemical composition by GC–MS and HPLC–MS analyses. The strain showed strong antimicrobial activity against *Staphylococcus aureus*, *Bacillus subtilis*, and *Candida albicans*, with minimum inhibitory concentrations (MICs) ranging from 62.5 to 250 µg/mL. Molecular identification using 16S rRNA gene sequencing and scanning electron microscopy confirmed the affiliation of the isolate to the genus *Streptomyces*. GC–MS analysis of the ethyl acetate extract revealed a complex mixture of volatile organic compounds, including fatty acid methyl esters, long-chain hydrocarbons, and sesquiterpenes, many of which are associated with antimicrobial, antioxidant, or anti-inflammatory properties. Complementary HPLC–MS profiling identified more than 40 nonvolatile bioactive compounds, including macrolides (erythromycin), polyethers (nigericin), aminoglycosides (netilmicin), polyketides (cytosporone C), and various sugar and lipid derivatives. The chemical diversity of the extract highlights the rich biosynthetic capacity of BPA-6 and its potential as a source of novel natural products.

**Supplementary Information:**

The online version contains supplementary material available at 10.1007/s11306-026-02427-3.

## Introduction

Antimicrobial resistance (AMR) has escalated into a major global health threat, undermining the efficacy of treatments across human and veterinary medicine (World Health Organization 2025; Tacconelli et al., 2018). The widespread overuse and misuse of antibiotics in healthcare and agriculture, spontaneous genetic mutations, inadequate sanitation, and the overuse use of biocides, has accelerated the emergence and dissemination of multi-drug-resistant pathogens (O’Neill, 2016; Aslam et al. 2018). Recent estimates suggest that AMR could cause up to 10 million deaths annually by 2050 if novel interventions are not developed (Naghaviet al., 2024).

Natural product discovery remains a cornerstone in the search for new antimicrobials. Microbial secondary metabolites—encompassing polyketides, non-ribosomal peptides, alkaloids, and terpenoids—have yielded the majority of clinically used antibiotics (Lee et al. 2020; Li et al. 2023). Actinomycetes, and in particular the genus *Streptomyces*, are renowned for their prolific biosynthetic capacity: over 60% of known microbial secondary metabolites originate from these filamentous bacteria (Djinni et al., 2019; Kumar et al., 2024). Cutting-edge analytical techniques such as UHPLC–HRMS, and GC–MS have further expanded our ability to profile complex metabolomes and to uncover cryptic biosynthetic pathways (Passari et al., 2017).

Bee-derived matrices have recently attracted attention as underexplored reservoirs of bioactive actinomycetes. High-throughput sequencing studies have identified *Streptomyces* spp. as dominant taxa in bee pollen and bee bread, where they may produce unique compounds shaped by the hive microenvironment (Shi et al. 2025; Saravana Kumar et al., 2014; Ghosh et al., 2022). These findings suggest that bee pollen is a promising niche for isolating novel *Streptomyces* strains with potent antimicrobial potential.

In this study, we isolated a *Streptomyces* sp. strain BPA-6 from Algerian bee pollen and applied GC–MS and UHPLC–MS to comprehensively characterize its secondary metabolite profile. We also assessed the antimicrobial activity of the obtained extracts against a panel of relevant microbial pathogens, aiming to identify candidate compounds for future drug development.

## Materials and methods

### Reagents, media, and microorganisms

Dimethyl sulfoxide (DMSO) and yeast extract were purchased from Sigma. Glycerol and ethyl acetate were purchased from Merck. Tetrazolium salt (0.25%)was from MicroBiotech. Muller-Hinton broth was obtained from Conda and malt extract was obtained from Liofilchem. The GF-1 Nucleic Acid Extraction Kit and universal primers (27F and 1492R) were from VivantisTechnologies.Gentamycin discs were provided from Oxoid™. *Staphylococcus aureus* ATCC 6538P, *Bacillus subtilis* ATCC 6633, *Escherichia coli* ATCC 7839, *Pseudomonas aeruginosa* ATCC 27853, *Klebsiella pneumoniae* ATCC 13883, and *Candida albicans* ATCC 10231 were obtained from the applied microbiologylaboratory (Ferhat Abbas University, Algeria).

### Characterization of the actinobacterial strain (BPA-6)

The actinobacterial strain, BPA-6, used in this study was obtained from the isolate collection of the applied microbiology laboratory (University of Ferhat Abbas Setif 1, Algeria), that were isolated from bee pollen samples in 2020.The strain was subcultured, purified, and maintained in slant culture on 15% glycerol yeast extract-malt extract agar (ISP-2) and stored at 4 °C as stock culture.

#### Morphological, cultural, and biochemical characteristics

The BPA-6 strain was initially characterized based on macroscopic and microscopic properties, including growth abundance, colony texture,sporecolor, aerial and substrate mycelium color, soluble pigment, biochemical characters, Gram staining, and scanning electron microscopy (Pérez-Corral et al., 2022; Mehta and Jadeja et al., 2022).

To conduct scanning electron microscopy, a 14 × 14 mm agar block with an active culture of BPA-6 from ISP2 media was placed into 2% glutaraldehyde in 0.2 M phosphate buffer (PBS, pH 7.2) and left for 48 h (24 h at 25 °C and 24 h at 4 °C). After that, the agar pieces were rinsed in three 30-min changes of PBS buffer, followed by a 60-min change in 30% ethanol, 50% ethanol, and 70% ethanol. The samples were examined with an electronic microscope EVO-50 (Carl Zeiss).

#### Molecular identification

The BPA-6strain was molecularly identified by the Gene Life Sciences Laboratory (Sidi Bel Abbes, Algeria) as a paid service. Briefly, the DNA was extracted using the GF-1 Nucleic Acid Extraction Kit (Vivantis Technologies, Malaysia), following the manufacturer's instructions. Then, 27F and 1492R primers were used to amplify the 16S rRNA under the following PCR conditions: initial denaturation at 94 °C for 12 min, denaturation at 94 °C for 30 s, annealing at 55 °C for 30 s, and extension at 72 °C for 1 min and 40 s. The amplification process was repeated for 30 cycles, followed by a final extension at 72 °C for 7 min. After that, the PCR products were subjected to electrophoresis, purified, and subsequently sequenced with the help of an automated DNA sequencer (Applied Biosystems®, USA) (Kamil et al., 2014). After sequencing, The Basic Local Alignment Tool (BLAST) program was used to compare the obtained sequenceof the 16S rRNA gene of our strain (BPA-6) with eight homologous sequences available in the NCBI GenBank database (Table 1), and subsequently the phylogenetic analysis was performed using MEGA 10 software constructed A phylogenetic tree was using neighbour-joining with 1000 bootstrap replicates (Song et al., 2004).

**Table 1:** Morphological, cultural, and biochemical properties of the BPA-6 strain

Properties	The isolate (BPA-6)
Morphological and cultural characteristics	
Growth abundance	Abundant growth
Growth texture	Powdery
Spore mass color	Light yellow
Aerial mycelium color	White
Substrate mass color	Dark yellow
Gram staining	Positive
Shape	Filamentous
Diffusible pigment	Negative
Carbon and nitrogen sources assimilation	
Glucose	+
Fructose	+
Maltose	−
Mannose	+
Arabinose	−
Saccharose	+
Galactose	+
Glycine	−
Proline	+
Arginine	−
Enzymes production	
Catalase	+
Oxidase	−
Protease	+
Lipase	+
Amylase	−

### Production and extraction of crude extract

Firstly, the strain BPA-6 was cultured on ISP-2 agar for 7 days at 28 °C. Then, with the help of a sterile cork borer, a 2 cm^2^ agar disc was obtained from pure culture and inoculated into an Erlenmeyer flask containing 100 mL of sterile ISP-2 broth, which was subsequently incubated at 28 °C ± 2 °C in an orbital shaker (200 rpm) for 7 days. After that, the mycelia were removed from the resulting culture broth by filtration through a filter paper, followed by centrifugation at 4000 rpm and 4 °C for 10 min. The cell-free clear filtrate (supernatant) was divided into three parts: the first part was filtered through a 0.45 µm syringe filter and kept directly at 4 °C for the antimicrobial activity screening. The secondwas extracted by an equal volume of pure ethyl acetate, evaporated, dissolved in 20% DMSO, filtered through a 0.45 µm syringe filter, and conserved at 4 °C for GC–MS analyses and antimicrobial activity, while the third part was lyophilized and subjected to UHPLC-HRMS/MS analyses (Al-Dhabi et al., 2019; Osama et al., 2022; Bhattarai et al., 2022).

### Antimicrobial activity

#### Perpendicular streak method

The perpendicular streak method was used to evaluate the primary screening for antimicrobial activity of BPA-6 against some pathogenic microorganisms, including *S. aureus* ATCC 6538P, *B. subtilis* ATCC 6633, *E. coli* ATCC 7839, *P. aeruginosa* ATCC 27853, *K. pneumoniae* ATCC 13883, and *C. albicans* ATCC 10231 (Singh et al., 2016). Briefly, a sterile inoculation loop was used to streak the BPA-6 strain in a straight line across the surface of a Mueller-Hintonagar plate at the center. The plate was then incubated for 5 to 7 days at 28 °C, allowing any produced antimicrobial compounds to diffuse into the agar medium. After the incubation period, all pathogenic microorganisms were adjusted to 10^7^ CFU/mLand streaked in separate straight lines, intersecting the central streak at a 90-degree angle. The plate was once again incubated for 18 to 24 h at 37 °C, and observations were recorded to evaluate the presence of growth or inhibition at the intersection of straight lines and the central streak. As a result, if the strain BPA-6 produces antimicrobial compounds, they will diffuse into the surrounding agar medium, inhibiting the growth of the test microorganisms. Therefore, the formation of clear inhibition zones at the intersections is a positive outcome.

#### Agar well diffusion method

As a secondary screening, the antimicrobial activities of the cell-free clear filtrate and the crude ethyl acetate extract, obtained from the resulting culture broth after submerged fermentation of the BPA-6 strain, were evaluated on Muller-Hinton agar plates using the agar well diffusion method against the previously tested pathogenic microorganisms (Balouiri et al., 2016). Prepared suspensions of 10^7^ CFU/mLfrom the young cultures of tested pathogenicmicroorganisms were swabbed on Muller-Hinton agar plates. Then, 100 mL from the cell-free clear filtrate and the crude ethyl acetate extract were poured into wells that were made using sterilized micropipette tips.All plates were incubated for 18 to 24 h at 37 °C. Gentamycin was used as a positive control. The inhibition zone diameter observed around each well was measured and is expressed in millimeters(mm). All experiments were carried out in triplicate. The results were expressed as means ± standard deviations.

#### Determination of minimum inhibition concentration (MIC)

With little modification, the microbroth dilution method in a 96-well microplate was used to determine the minimum inhibitory concentration (MIC) of the crude ethyl acetate extract of the actinobacterial strain (BPA-6) (Balouiri et al., 2016; Kadeřábková et al., 2024). 160 µL of sterile Mueller–Hinton broth was transferred to each well of the microplate, followed by the addition of 20 µL of 10^7^ CFU/mLfrom young culture suspensions of *S. aureus* ATCC 6538P, *B. subtilis* ATCC 6633, and *C. albicans* ATCC 10231. Then, 20 µL of different concentrations of the crude ethyl acetate extract from 7.81 to 1000 µg/mLwere added to the required wells. Gentamycin and sterile Mueller–Hinton broth were used as positive and negative controls, respectively. After then, all microplates were incubated at 37 °C for about 24 to 48 h. Finally, 20 µL of 0.25%tetrazolium salts (TTC) was added to wells of microplates as an effective growth indicator and incubated in dark conditions for 30 min. As a result, the first concentration at which it gave no color change was considered the MIC value. All experiments were performed in triplicate.

#### Minimum microbicidal concentration (MMC)

The minimum microbicidal concentration (MMC)was determined directly from the MIC results priorto the addition of 0.25%tetrazolium salts (TTC) (Balouiri et al., 2016; Kadeřábková et al., 2024).With the help of a bacteriological platinum handle, a loop from each well of 96-well microplates was streaked on Mueller–Hinton agar plates and incubated for 24 h at 37 °C. The lowest concentration of the crude ethyl acetate extract that showed no visible microbial growth was recorded as the MMC value. All experiments were performed in triplicate.

### GC–MS analysis

The crude ethyl acetate extract was dissolved in DMSO, filtered through a 0.22 µm syringe filter, and injected for direct analysis by the GC–MS Shimadzu QP-2030 system equipped with an Rxi-5 ms capillary column (length 30 m, diameter 0.25 mm, and film thickness 0.25 µm). The obtained MS spectrum was interpreted by comparing it with the National Institute of Standards and Technology database to determine the bioactive compounds produced by the strain (BPA-6) with names, retention times, amounts, and suggested chemical structures (Cunha et al., 2024; Kumar et al., 2024).

### UHPLC-HRMS/MS analyses

#### Sample preparation

An aliquot of 30 mg from the extract obtained from the A6 strain was dissolved in 2 mL of water. The sample was sonicated for 15 min and centrifugated at 14,000 rpm at 4 °C for 10 min. Then the supernatant was filtered with 0.2 µm.

#### UHPLC-HRMS/MS analysis

To evaluate the presence of compounds in the extracted samples A6, the Orbitrap IQ-X mass spectrometer coupled with Vanquish UHPLC system from Thermo Fisher Scientific (Waltham, Massachusetts, USA) was employed to provide a detailed mass analysis. This allowed an in-depth investigation into the mass spectra of each compound, supporting the identification of compounds with high resolution and accuracy.

Compounds were separated by BEH C18 column (150.0 mm × 2.1 mm, particle size 1.7 µm) from Waters Corporation (Milford, Massachusetts, USA) equipped with a VanGuard C18 guard column (5.0 mm × 2.1 mm, particle size 1.7 µm). The flow rate was set at 300 µL min^−1^ at 45 °C. H_2_O was used as eluant A and acetonitryle (CAN) as eluant B, and both were acidified with 0.1% formic acid. The chromatographic system operated at a flow rate of 0.400 mL min^−1^ with a 2 µL sample injection volume. Analytes were eluted using a gradient program that started with 5% phase B for 1 min, increased to 100% B at 11 min, reached 100% B at 4 min, and returned to the starting conditions within 0.5 min., reached at 4.5 min with a total run time of 19 min.

Ionization was obtained by Heated ElectroSpray Ionization (H-ESI) operating in positive and negative mode. Nitrogen was used as sheath gas and auxiliary gas. HESI default settings were used based on the UPLC flow. The ion transfer tube temperature was 325 °C; the source heater temperature was 300 °C; and the source voltage was 2.5 kV. Before analysis, the accurate mass was calibrated using the Pierce FlexMix Calibration Solution for Auto-Ready calibration (Thermo Scientific). Fluoranthene was used as the internal EASY-IC calibrant for full MS spectra. Full MS data were collected in positive and negative ionization modes over the *m/z* range of 100–1000 at 30,000 full widths at half maximum (FWHM) resolution.

MS^2^ analysis was performed using HCD with energy set at 25 eV and mass tolerance was set at 5 ppm. The mass resolutions for MS^2^ spectra were set at 30,000 FWHM. The data were processed using Freestyle version 1.8 (Thermo Fisher Scientific). Theoretical masses were calculated using the Scientific Instrument Services (SIS) website.

A metabolomics workflow was designed for untargeted analysis, utilizing the Compound Discoverer™ version 3.3 software (Thermo Fisher Scientific). Initially, the files (raw files) were loaded into the software. Procedure blanks were included in the workflow to facilitate background signal removal and area normalization, respectively, with blanks labeled as “Blank”. The workflow commenced with compound detection through feature extraction in the retention time (RT) and mass-to-charge (m/z) ranges of 0–20 min and 100–1000, respectively, covering both HESI + and HESI- polarities. Identified analytes were then matched to LC–MS spectral libraries for compound identification, using databases aligned with the sample type, including ChemSpider, Natural Products Atlas, HMDB, FooDB,MassBank, PubMed, KEGG, ChEBI, ChemBank and mzCloud, with a mass tolerance of 5 ppm for high-precision annotation.

Furthermore, metabolite annotation was performed based on accurate mass measurements and MS/MS spectral matching against public databases. The resulting assignments should be considered putative annotations rather than definitive identifications unless confirmed with authentic standards.

### Data analysis

The antagonism activity was expressed as means ± standard deviation (SD). The means were compared using an analysis of variance (ANOVA one-way), followed by the Fisher test (*P* = 0.05). The statistical analyses were performed with the GraphPad 8.0 program (GraphPad Prism).

## Results

### Morphological, cultural, and physiological characteristics of the isolate (BPA-6)

**Fig. 1:**
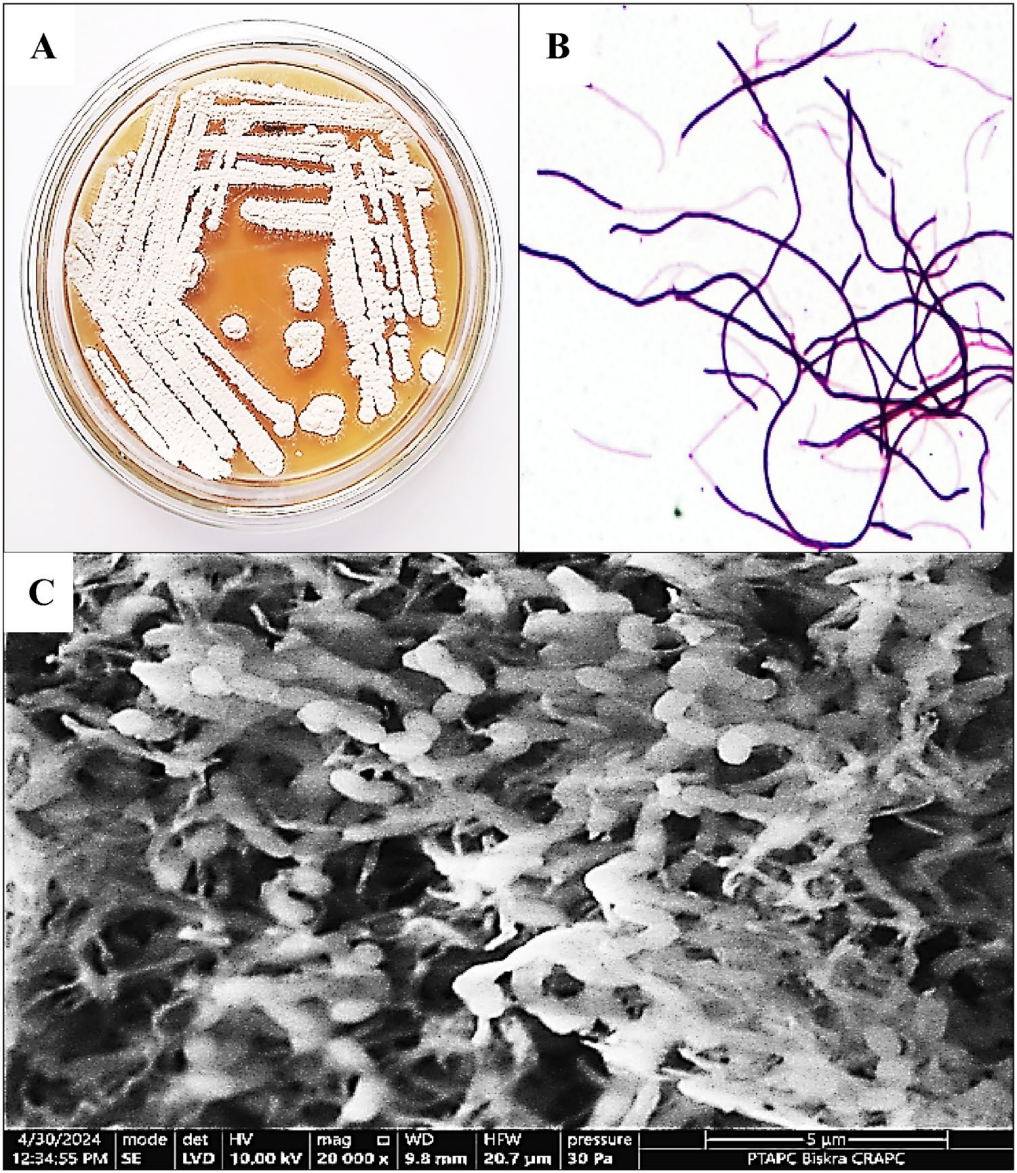
Morphological and cultural properties of the BPA-6 strain. **A** colonies of BPA-6 strain grown on ISP-2 agar, **B** Gram staining micrograph, **C** Scanning electron micrograph showing spore morphology of *Streptonyces* sp. BPA-6

The actinobacterial isolate BPA-6 exhibited hallmark *Streptomyces* morphology and robust cultural characteristics on ISP-2 agar (Table 1). Macroscopically, the colony displayed abundant powdery growth, indicative of prolific aerial mycelium formation. The spore mass was light yellow, contrasting with the dark yellow substrate mycelium, while the aerial mycelium appeared white. This coloring pattern—light‐yellow spores over a darker substrate—reflects typical pigment partitioning in many soil‐derived *Streptomyces* species. Microscopic examination confirmed filamentous, Gram-positive hyphae (Fig. 1B) without diffusible pigment production, further aligning BPA-6 with classical *Streptomyces* taxonomy.

Additionally, the SEM image (Fig. 1C) of *Streptomyces* sp. BPA 6 reveals a complex network of intertwined hyphae and spore chains. It appears as branched, rod-like structures, typically 0.5–1 µm in diameter, tightly packed, and often crossed, characteristic of mature aerial mycelial architecture. Notably, the spore’s chains exhibit a straight or slightly curved arrangement, with spores closely spaced along the hyphae. In addition, the high-resolution details show clean, smooth spore surfaces, lacking ornamentation or apparent depressions, common features of many soil *Streptomyces*. The visible septa along the aerial hyphae result from mature sporulation stages, during which layers of hydrophobic rods have formed to promote spore differentiation and dispersal.

Biochemical profiling revealed a selective yet versatile substrate utilization pattern (Table 1). BPA-6 efficiently assimilated glucose, fructose, mannose, sucrose, galactose, and proline, demonstrating capacity to metabolize both hexose sugars and amino acids. In contrast, the isolate failed to utilize maltose, arabinose, glycine, and arginine, suggesting absence or low activity of specific uptake systems or catabolic enzymes for these substrates. This assimilation fingerprint implies that BPA-6 binds preferentially on common pollen carbohydrates—particularly monosaccharides and certain disaccharides—and specific amino acids for growth.

Enzymatic assays highlighted BPA-6’s potential for extracellular hydrolytic activity. The strain was catalase-positive, confirming effective breakdown of hydrogen peroxide, and protease- and lipase-positive, indicating secretion of enzymes capable of degrading proteinaceous and lipid substrates in its environment. The absence of oxidase activity suggests a respiratory chain lacking cytochrome-c-oxidase, typical of many *Streptomyces*. Notably, BPA-6 did not produce amylase, consistent with its inability to hydrolyze maltose, and correlating with the negative assimilation of this disaccharide substrate.

Collectively, these morphological and biochemical traits portray BPA-6 as a classical *Streptomyces* with strong aerial development, pigment differentiation, and selective metabolic capabilities. Its proficiency in proteolytic and lipolytic enzyme production, combined with a preference for common pollen sugars and specific amino acids.

### Molecular identification of the isolate BPA-6

**Fig. 2:**
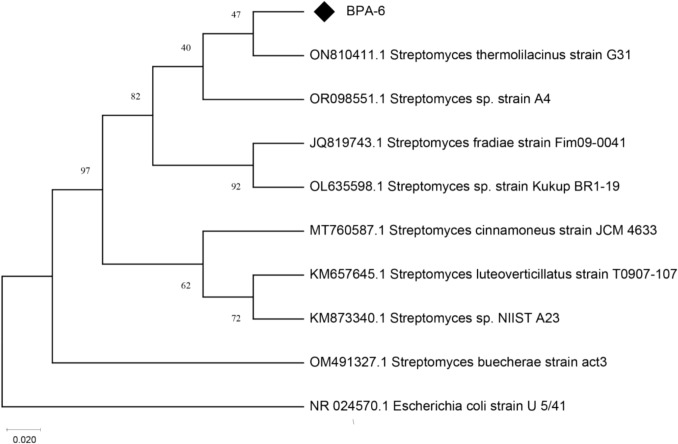
Neighbor-joining phylogenetic tree using the Maximum Likelihood method based on 16S rRNA gene sequences of the BPA-6 strain

The 16S rRNA gene sequence of *Streptomyces* sp. BPA-6 was aligned by BLAST with eight *Streptomyces* strains sequenced in the NCBI GenBank database. As summarized in Table 2, BPA-6 displayed 100% query coverage and an E-value of 0.0 for all hits, with the highest pairwise identity being 90.24% with *Streptomyces* sp. A4 (OR098551.1), followed by 89.11% with *S. thermolilacinus* strain G31 (ON810411.1). The lowest identities were between 88.5 and 89.1% among other *Streptomyces* species, including *S. luteoverticillatus*, *S. cinnamoneus*, *S. buecherae*, *S. fradiae*, and *Streptomyces* sp. Kukup (88.54–89.14%). A neighbor-joining phylogenetic tree (Fig. 2) placed BPA-6 within the genus *Streptomyces*, but as a deeply divergent lineage with no close neighbors above the species-level shared identity threshold (≥ 98.7%).

**Table 2:** Homologous sequences to the BPA-6 strain available in the GenBank database

No	Description	Query cover (%)	E value	Pairwise Identity (%)	Accession number
1	*Streptomyces* sp. strain A4	100	0.0	90.24	OR098551.1
2	*Streptomyces thermolilacinus* strain G31	100	0.0	89.11	ON810411.1
3	*Streptomyces* sp. NIIST A23	100	0.0	89.09	KM873340.1
4	*Streptomyces luteoverticillatus* strain T0907-107	99	0.0	89.14	KM657645.1
5	*Streptomyces cinnamoneus* strain JCM 4633	99	0.0	88.98	MT760587.1
6	*Streptomyces buecherae* strain act3	99	0.0	88.98	OM491327.1
7	*Streptomyces fradiae* strain Fim09-0041	99	0.0	88.83	JQ819743.1
8	*Streptomyces* sp. Strain Kukup BR1-19	99	0.0	88.54	OL635598.1

### Antimicrobial activity

#### Perpendicular streak method

**Fig. 3:**
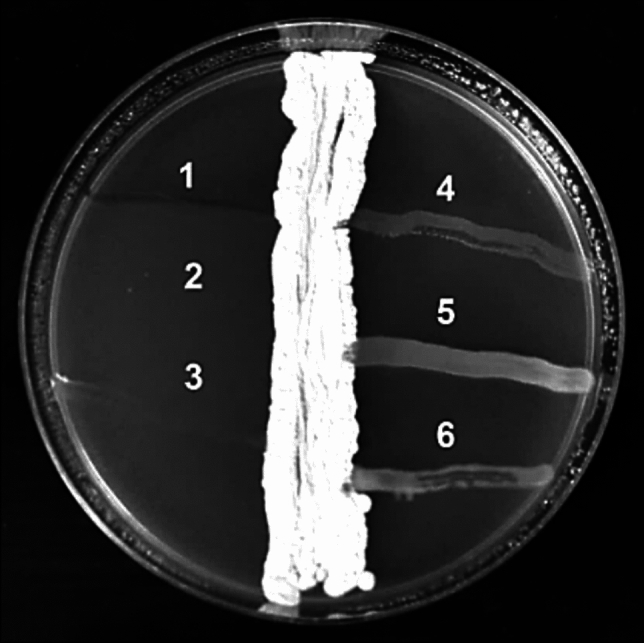
Primary screening of the *Streptomyces* sp. BPA-6 strain against (1) *S. aureus* ATCC 6538P, (2) *B. subtilis* ATCC 6633, 3) *C. albicans* ATCC 10231, (4) *E. coli* ATCC 7839, (5) *P. aeruginosa* ATCC 27853, 6) *K. pneumoniae* ATCC 13883

Based on the perpendicular streak method, the *Streptomyces* sp. BPA-6 strain was subjected to primary screening for antimicrobial activity to determine its ability to secrete antimicrobial secondary metabolites against several pathogenic microorganisms, including *S. aureus* ATCC 6538P, *B. subtilis* ATCC 6633, *E. coli* ATCC 7839, *P. aeruginosa* ATCC 27853, *K. pneumoniae* ATCC 13883, and *C. albicans* ATCC 10231 (Fig. 3). According to the results, the BPA-6 strain displayed only antagonistic activity against *S. aureus* ATCC 6538P, *B. subtilis* ATCC 6633, and *C. albicans* ATCC 10231.

##### Agar well diffusion method

**Fig. 4:**
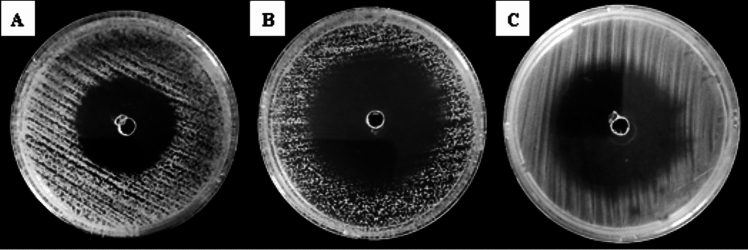
Secondary screening for antimicrobial activity of the crude ethyl acetate extract of the *Streptomyces* sp. BPA-6 strain against: **A** *B. subtilis* ATCC 6633, ** B** *C. albicans* ATCC 10231, **C** *S. aureus*ATCC 6538P

In this study, the culture supernatant (clear filtrate) and the crude ethyl acetate extract of the *Streptomyces* sp. BPA-6 strain were also tested against *S. aureus* ATCC 6538P, *B. subtilis* ATCC 6633, *E. coli* ATCC 7839, *P. aeruginosa* ATCC 27853, *K. pneumoniae* ATCC 13883, and *C. albicans* ATCC 10231 using the agar well diffusion method as a secondary screening for antimicrobial activity (Table 3, Fig. 4).

**Table 3:** Agar well diffusion method results of the crude ethyl acetate extract of the *Streptomyces* sp. BPA-6 strain

Test microorganisms	Inhibition zones (mm)		
	Supernatant	Ethyl acetate extract	Gentamycin
*S. aureus* ATCC 6538P	30.33 ± 0.94	42.33 ± 3.79	28
*B. subtilis* ATCC 6633	33.33 ± 1.53	41.66 ± 3.51	36
*E. coli* ATCC 7839	00	00	21
*K. pneumoniae ATCC 13883*	00	00	27
*P. aeruginosa* ATCC 27853	00	00	21
*C. albicans* ATCC 10231	33.00 ± 1.41	48.67 ± 2.08	/

As shown in Table 3, the culture supernatant of the *Streptomyces* sp. BPA-6 strain showed a positive antimicrobial activity that corresponds to the results of the primary screening against *S. aureus* ATCC 6538P (30.33 ± 0.94 mm), *B. subtilis* ATCC 6633 (33.33 ± 1.53 mm), and *C. albicans* ATCC 10231 (33.00 ± 1.41 mm).And as expected, the crude ethyl acetate extract was stronger than the standard antibiotic against*S. aureus* ATCC 6538P, *B. subtilis* ATCC 6633, and *C. albicans* ATCC 10231, with inhibition zones of 42.33 ± 3.79, 41.66 ± 3.51, and 48.67 ± 2.08 mm, respectively.

#### Determination of MICs and MMCs

The minimum inhibitory concentrations (MICs) and the minimum microbicidal concentrations (MMCs) of the crude ethyl acetate extract of the *Streptomyces* sp. BPA-6 strain against *S. aureus* ATCC 6538P, *B. subtilis* ATCC 6633, and *C. albicans* ATCC 10231were evaluated using the microbroth dilution method in a 96-well microplate with the help of tetrazolium salts (TTC) as an effective growth indicator and subculture technique.

The MICs and MMCs of the crude ethyl acetate extract of *Streptomyces* BPA-6 were determined successfully and were presented in Table 4. The recorded MIC value was 62.5 µg/mL against *S. aureus* ATCC 6538P and *B. subtilis* ATCC 6633, while it was 250 µg/mL against *C. albicans* ATCC 10231. These results indicate that the crude ethyl acetate extract of the *Streptomyces* BPA-6 strain contains bioactive secondary metabolites that can be used as antibiotics to inhibit the growth of pathogenic microorganisms, particularly against *B. subtilis* ATCC 6633, with an MMC value of 1000 µg/mL.

**Table 4:** MICs and MMCs results of the crude ethyl acetate extract of the *Streptomyces* sp. strain BPA-6

Test microorganisms	MIC (µg/mL)	MMC (µg/mL)
*S. aureus* ATCC 6538P	62.5	> 1000
*B. subtilis* ATCC 6633	62.5	1000
*C. albicans* ATCC 10231	250	> 1000

### GC–MS analysis

The GC–MS analysis of the ethyl acetate extract of *Streptomyces* sp. BPA-6 revealed a chemically diverse metabolite profile comprising 43 identifiable compounds (Table 5), which spanned across multiple structural classes, including fatty acid esters, diterpenes, sesquiterpenes, triterpenes, phenols, phenanthrenes, and sterols. Notably, the extract was dominated by the oxygenated sesquiterpenoid 4-isopropyl-1,7,11-trimethyl-2,7,11-cyclotetradecatrien-1-ol, which accounted for 28.90% of the total ion chromatogram area. This compound, often associated with quorum sensing inhibition and membrane-targeting antibacterial effects, is a hallmark of bioactive microbial secondary metabolites, particularly those from actinobacteria. Other significant contributors included hexadecanoic acid methyl ester (11.33%) and unsaturated fatty acid esters such as methyl linoleate and methyl oleate, which collectively enhance the antimicrobial potency via surfactant-like membrane disruption and inhibition of phospholipase A2 activity. The detection of phenanthrene derivatives (e.g., compounds 19 and 30) and rare diterpenoids (e.g., compound 10 and 29) underlines the strain’s capacity to produce structurally complex scaffolds with potential cytotoxic and anti-inflammatory properties, paralleling compounds isolated from higher plants and marine microbes.

**Table 5:**
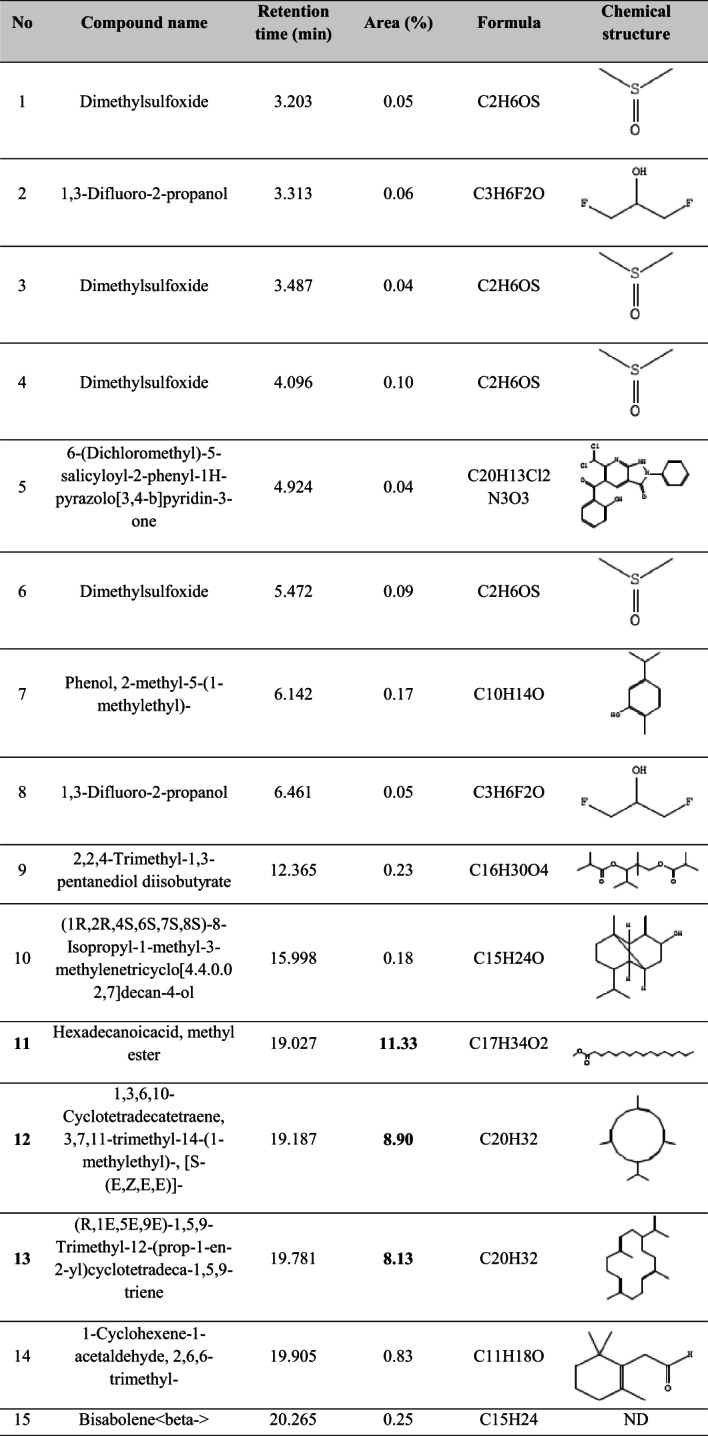

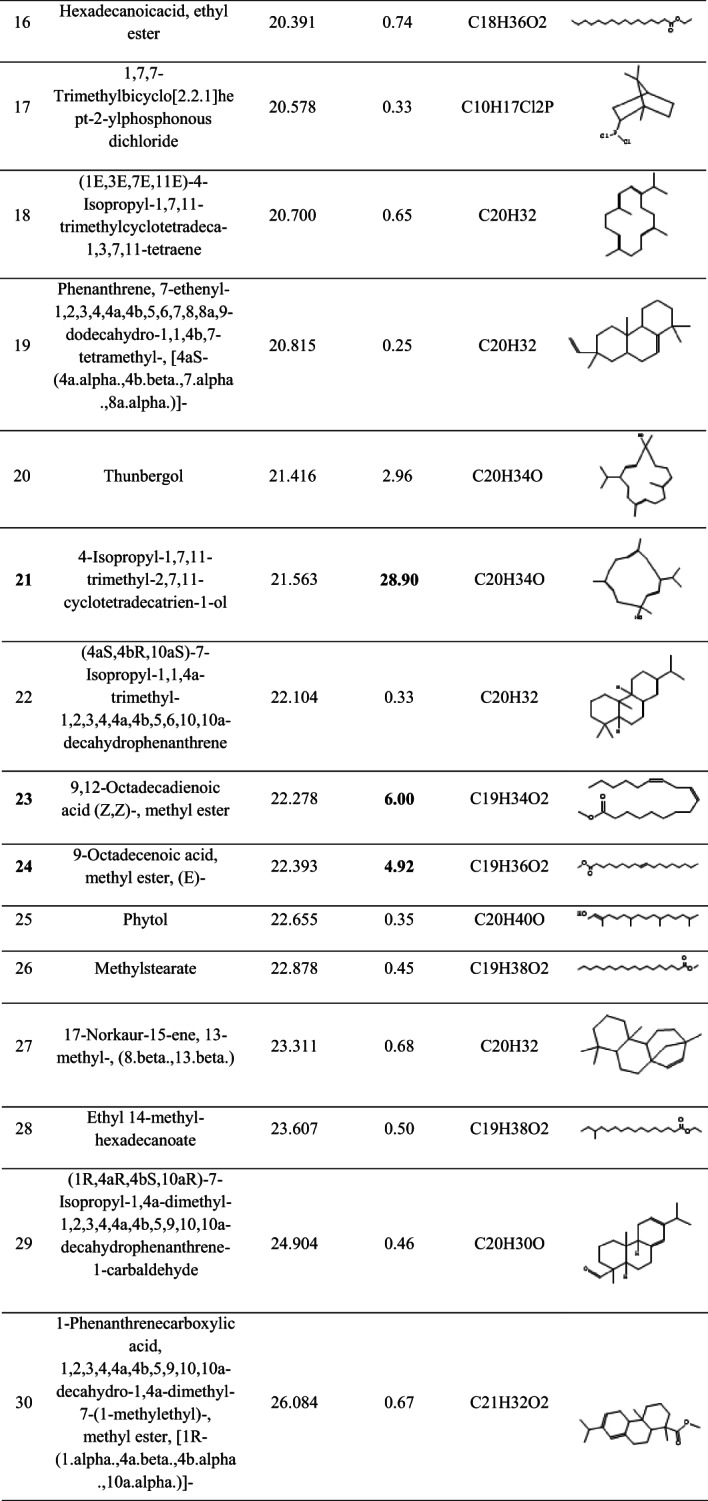

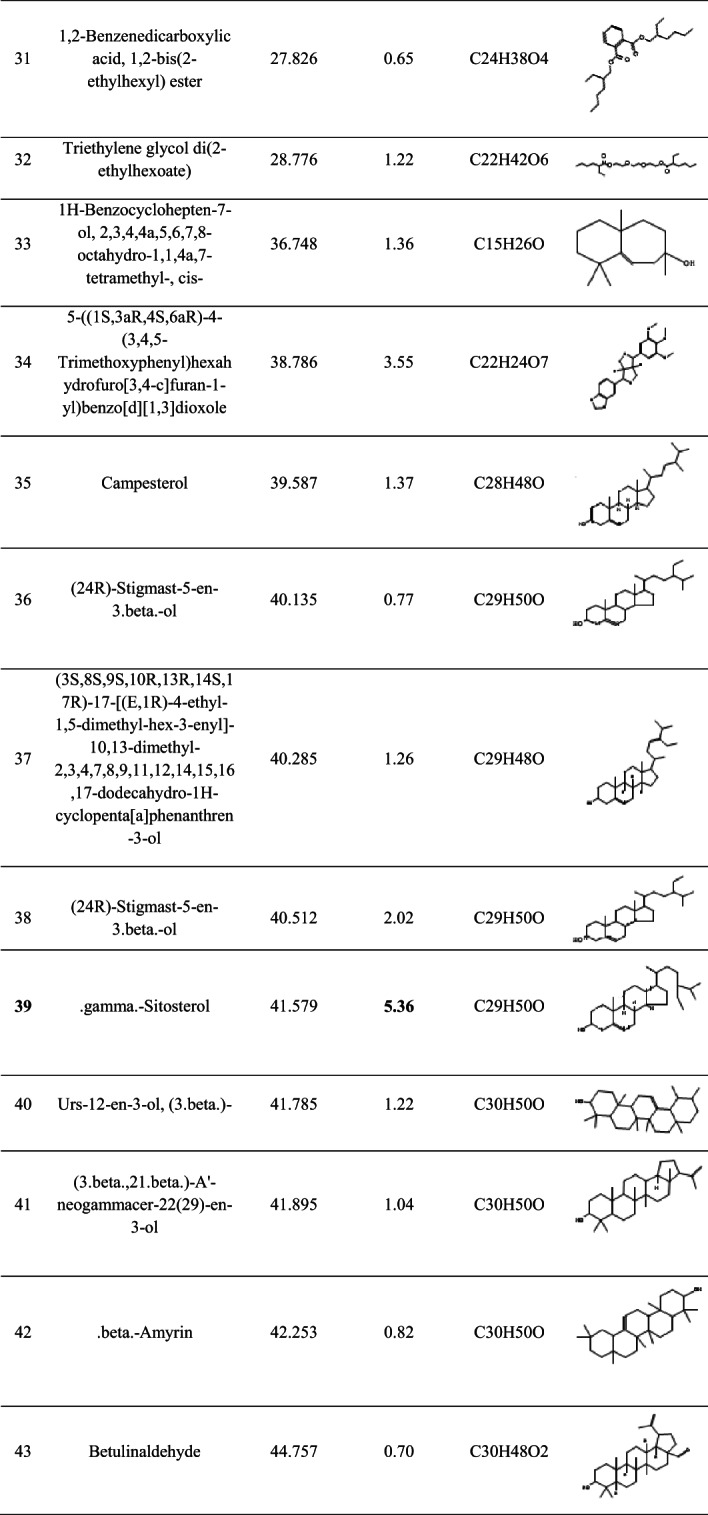
The bioactive compounds detected by GC–MS analysis in the crude ethyl acetate extract of the *Streptomyces* sp. strain BPA-6

Of particular chemotaxonomic interest is the occurrence of triterpenoids and phytosterols—such as β-amyrin, urs-12-en-3-ol, γ-sitosterol, and campesterol—in the extract, highlighting a possible horizontal gene transfer or endosymbiotic pathway, as these metabolites are typically associated with plant biosynthesis.

Several compounds have been reported in the literature to exhibit anticancer and anti-inflammatory activities and to modulate NF-κB signaling. However, these activities were not evaluated in the present study and are mentioned here only to highlight the potential biological relevance of the annotated metabolites.

### Metabolite profiling of *Streptomyces* sp. strain BPA-6 by UHPLC-HRM/MS

High-performance liquid chromatography coupled with mass spectrometry (HPLC–MS) was employed to analyze the metabolic profile of *Streptomyces* sp. strain BPA-6. Metabolite profiling was performed using UHPLC–HRMS/MS. Compound annotation was based on accurate mass measurements, isotopic distribution, and comparison of MS/MS fragmentation patterns with publicly available spectral libraries and previously reported literature data. Candidate metabolites were assigned when the mass error between the experimental and theoretical m/z values was below 5 ppm and when characteristic fragment ions matched those reported in reference databases. A total of 24 compounds were identified based on their retention times, accurate mass-to-charge ratios (m/z), and diagnostic fragment ions in both positive and negative ionization modes (Table 6). These metabolites reflect the strain’s ability to produce a wide range of primary and secondary metabolites with diverse bioactivities. Furthermore, Putative identification of several bioactive metabolites, including Erythromycin, Netilmicin, and Nigericin, was achieved through spectral matching. Authentic analytical standards were not available for all detected compounds; therefore, retention time confirmation was not performed. To reduce the possibility of false-positive annotations, only metabolites meeting strict criteria of mass accuracy, isotopic pattern agreement, and diagnostic MS/MS fragment consistency were retained.

**Table 6:** UHPLC-HRMS-MS analysis for the chemical composition of ethyl acetate extract of *Streptomyces* sp. Strain BPA-6

Name	Formula	Calc. MW	m/z	RT [min]	Reference Ion	Fragments	In extract of BPA-6
L-valine	C5 H11 N O2	117,07886	118,08613	0,817	[M + H] + 1	118.0862, 72.08078,55.05423,	-
9'-deoxy-8',8'-dihydroxyherbicidin B	C18 H23 N5 O9	453,14,792	452,14,065	1,079	[M-H]-1	134.04722, 140.09417, 206.06835, 92.02542	-
α-L-Rhap-(1- > 4)-D-ribitol-5-phosphate	C11 H23 O12 P	378,09274	377,08546	1,78	[M-H]-1	219.08741,163.0612, 89.02442, 71.01385	+
4-[5-Hydroxy-6-(2-hydroxy-3-methyl-4-methylenetetrahydro-2-furanyl)-1,3-dioxan-4-yl]-2,6-piperidinedione	C15 H21 N O7	327,13,166	328,13,894	1,884	[M + H] + 1	310.12851, 128.0706, 97.06479	+
Raffinose	C18 H32 O16	504,16,898	503,1617	1,9	[M-H]-1	485.15119, 341.10894, 179.05611	+
Hexopyranosyl-(1- > 4)-[hexopyranosyl-(1- > 6)] hexopyranosyl-(1- > 4)hexopyranose	C24 H42 O21	666,22,168	665,21,441	2,01	[M-H]-1	575.18289, 503.16176, 179.05611	+
N-{3-[3-(1-piperidinomethyl) phenoxy]} propylamine	C15 H24 N2 O	248,18,874	249,19,602	2,245	[M + H] + 1	232.16959, 147.08044, 98.09643	+
β-D-Allopyranose	C6 H12 O6	180,06325	179,05597	2,8	[M-H]-1	161.04555, 147.0299, 73.0295	+
D-3-Deoxyglucosone	C6 H10 O5	162,05272	161,04544	3,7	[M-H]-1	143.03498, 131.03498,71.01385	+
N-acetylglycyl-L-ornithyl-L-leucinamide	C15 H29 N5 O4	343,22,052	344,2278	3,755	[M + H] + 1	177.11203, 133.08586, 89.05961	+
N-Acetyl-L-seryl-L-lysyl-L-leucinamide	C17 H33 N5 O5	387,24,643	388,25,371	3,901	[M + H] + 1	283.17514, 221.13831, 133.08592	+
Galactinol	C12 H22 O11	342,11,606	341,10,878	4	[M-H]-1	323.09837, 221.06668, 59.01385	+
O-(Aminomethyl)-L-homoseryl-L-leucyl-L-leucylglycine	C19 H37 N5 O6	431,27,248	432,27,976	4,05	[M + H] + 1	327.20123, 177.11198, 133.08580	+
Netilmicin	C21 H41 N5 O7	475,29,871	476,30,598	4,181	[M + H] + 1	441.27076, 257.19720, 127.08658	+
L-Isoleucyl-L-leucyl-L-α-aspartyl-L-valine	C21 H38 N4 O7	458,27,221	497,23,536	4,182	[M + K] + 1	399.27411, 458.34761	+
2-[(3 s,5 s,7 s)-Adamantan-1-yl]ethyl 6-deoxy-1-thiohexopyranoside	C18 H30 O4 S	342,18,625	341,17,898	7,099	[M-H]-1	297.20747, 183.01183, 119.05005	+
3-(Tetradecanoylamino)-4-(trimethylammonio)butanoate	C21 H42 N2 O3	370,31,937	371,32,672	8,219	[M + H] + 1	268.26334, 188.43489, 85.0109	-
MG (0:0/15:0/0:0)	C18H36O4	316.26114	315.2586	8.39	[M + H]-1	171.13867, 127.14922, 59.0138	+
Unclassified	C40 H69 N5 O7	731,52,083	732,52,811	8,419	[M + H] + 1	618.7543, 508.82620, 273.18771	+
Methyl N-[11-(dimethylamino)undecanoyl]-L-seryl-L-lysyl-L-leucinate	C29 H57 N5 O6	571,42,956	572,43,684	8,643	[M + H] + 1	512.41703, 301.22342, 129.10224	+
PC(20:5(5Z,8Z,11Z,14Z,17Z)/P-18:1(11Z))	C46 H80 N O7 P	789,56,359	790,57,086	8,651	[M + H] + 1	184.07332, 124.99982, 99.11683	-
(9Z)-N-Methyloctadec-9-enamide	C19 H37 N O	295,28,732	296,2946	9,339	[M + H] + 1	265.25259, 209.22637,125.13247	+
Cytosporone C	C16 H22 O4	278,15,159	279,15,886	9,829	[M + H] + 1	149.02342, 121.03977, 47.06990	+
N-(11-Aminoundecanoyl)-L-seryl-N-(2-cyclohexylethyl)-L-lysinamide	C28 H55 N5 O4	525,42,406	526,43,134	9,913	[M + H] + 1	327.2014, 177.11195, 133.08578	-
2-[(2-acetamido-3-methylbutanoyl)-methylamino]-N-[(Z)-2-(1H-indol-3-yl) ethenyl]-4-methylpentanamide	C24 H34 N4 O3	426,26,505	425,25,777	10,747	[M-H]-1	355.14716, 211.02791, 122.97550	+
N ~ 2 ~ -[2-(Dimethylamino) ethyl]-N ~ 4 ~ -dodecyl-6-methyl-2,4-pyrimidinediamine	C21 H41 N5	363,33,461	364,34,189	10,748	[M + H] + 1	242.23392, 196.15567, 99.11683	+
Nigericin	C40 H68 O11	724,47,535	723,46,807	11,48	[M-H]-1	521.29413, 409.59332, 325.18359	+
Eritromicin	C37H67NO13	733.46124	734.46851	11,49	[M + H] + 1	716.45795, 558.36365, 158.11755	+
Tetracosanoic acid	C24 H48 O2	368,36,528	367,358	14,23	[M-H]-1	223.47964, 199.11963, 129.60464	+

According to the metabolite identification guidelines of the Metabolomics Standards Initiative, these annotations correspond to Level 2 (putatively annotated compounds).

Detected compounds such as β-D-allopyranose, D-3-deoxyglucosone, and raffinose are indicative of active primary metabolic processes. These molecules serve essential roles in amino acid biosynthesis, energy metabolism, and carbon storage. Although typically non-bioactive, such primary metabolites can act as precursors for secondary metabolite biosynthesis or modulate microbial interactions under environmental stress. Furthermore, several di- and tripeptide compounds were identified, including N-acetylglycyl-L-ornithyl-L-leucinamide, N-acetyl-L-seryl-L-lysyl-L-leucinamide, and O-(aminomethyl)-L-homoseryl-L-leucyl-L-leucylglycine. These low-molecular-weight peptides may exhibit antimicrobial or signaling properties, and some of them resemble known peptidomimetics. Also, the metabolomic analysis also revealed the presence of established antibiotics such as erythromycin, nigericin, and netilmicin. Erythromycin is a 14-membered macrolide that inhibits bacterial protein synthesis by targeting the 50S ribosomal subunit. Nigericin functions as a polyether ionophore, disrupting transmembrane ion gradients, while netilmicin is an aminoglycoside effective against Gram-negative pathogens. The co-occurrence of these compounds affirms the antibiotic-producing capacity of BPA-6 and its potential relevance in drug discovery.

Complex sugar derivatives such as galactinol, raffinose, and α-L-rhamnosyl-(1 → 4)-D-ribitol-5-phosphate were identified, suggesting roles in osmoprotection, cellular communication, or as metabolic intermediates. These compounds may also enhance probiotic growth or modulate host–microbe interactions, especially in soil environments. In addition, compounds including MG (0:0/15:0/0:0), tetracosanoic acid, and (9Z)-N-methyloctadec-9-enamide indicate lipid biosynthesis and membrane-related metabolic activity. These molecules can contribute to microbial fitness, antimicrobial tolerance, or even act as mild surface-active agents.

More importantly, several structurally intriguing metabolites were also detected. Cytosporone C, known for its anti-inflammatory and antitumor properties, was observed alongside an indole-containing pentanamide derivative and a pyrimidinediamine analog. These findings suggest the potential of BPA-6 to produce novel metabolites through cryptic or underexplored biosynthetic gene clusters.

## Discussion

### Phenotypic and genotypic identification

The morphological and cultural characteristics of *Streptomyces* sp. BPA-6 closely resembles those typically observed in terrestrial *Streptomyces* isolates. The powdery colony texture, abundant aerial mycelium, and pigmentation—light yellow spores over dark-yellow substrate mycelium—are consistent with descriptions of sporulating *Streptomyces* from environments (Pérez-Corral et al., 2022; Sharma et al., 2021). The white aerial mycelium and absence of diffusible pigment also align with diagnostic characteristics of several *Streptomyces* groups isolated from arid soils, known for their adaptation to harsh environments (Qin et al., 2016; Manikandan et al., 2017). The filamentous Gram-positive nature observed under microscopy further corroborates the taxonomic placement of BPA-6 within the genus *Streptomyces* (Goodfellow et al., 2012; Jadeja et al., 2022).

Carbon and nitrogen source assimilation profiles provided deeper insight into the metabolic specialization of BPA-6. The isolate readily utilized monosaccharides like glucose, fructose, mannose, and galactose, along with sucrose, but did not metabolize maltose or arabinose. This selective sugar utilization mirrors trends observed in other bioactive *Streptomyces* isolates, where strains show broad hexose metabolism but limited activity toward disaccharides or pentoses (Elsayed et al.,^ 2023^; Jadeja et al., 2022). The inability to hydrolyze maltose corresponds well with the observed lack of amylase activity, reflecting how extracellular enzyme production governs substrate assimilation (Berdy, 2012; Kalyani et al., 2019). The successful assimilation of proline but not glycine or arginine suggests the presence of specialized nitrogen uptake systems—adaptations frequently seen in soil actinomycetes responding to limited nitrogen pools (Shirling & Gottlieb, 1966). Proline is often abundant in pollen, making its catabolism ecologically advantageous (Khirennas et al., 2023).

Biochemical analysis confirmed catalase activity in BPA-6, which is typical among aerobic *Streptomyces* species and reflects their need to neutralize oxidative stress during secondary metabolism and sporulation (Liu et al., 2019). The production of extracellular protease and lipase further supports its ecological role in organic matter degradation, a hallmark of *Streptomyces* that contributes significantly to nutrient assimilation in ecosystems (Kalyani et al., 2019; Manivasagan et al., 2013). Lipases from *Streptomyces* are especially valued for their stability and potential applications in food and pharmaceutical industries (Arasu et al., 2021). The absence of oxidase activity is less common but not unprecedented and may reflect adaptations to microaerophilic niches or alternative respiratory pathways.

Collectively, these phenotypic traits affirm the initial identification of BPA-6 as a member of the *Streptomyces* genus. Its selective sugar and amino acid assimilation, enzyme production, and morphological characteristics highlight its potential for biotechnological applications, especially in environments where robust degradation of complex substrates is required. Furthermore, its ability to grow under simple nutritional conditions and secrete hydrolytic enzymes indicates a promising role in industrial enzyme production and perhaps even antimicrobial compound biosynthesis—traits often associated with *Streptomyces* from arid and semi-arid zones (Elsayed et al., 2023; Sharma et al., 2021).

The genus-level identity of *Streptomyces* sp.BPA-6 is well supported: published guidelines generally consider < 95% 16S rRNA gene similarity to distinguish between genes (e.g. < 95%). BPA-6’s identities of ~ 88–90% comfortably fall within the *Streptomyces* genus boundary (usually defined by ≥ 95% identity to known *Streptomyces* strains) (Johnson et al., 2019). However, species‑level assignment is precluded by the very low similarity (~ 89% vs the threshold of ≥ 98.7% consistent with accepted species‑level cutoff). Indeed, *Streptomyces* species often share > 99% identity and may still be genomically distinct, underscoring the low resolution of 16S rRNA alone for streptomycete species delineation (Komaki, 2023). Thus, we confirm BPA‑6 as a novel and distant lineage within the *Streptomyces* genus, warranting the temporary designation *Streptomyces* sp. until further characterization. For definitive species assignment, whole-genome sequencing to compute metrics such as average nucleotide identity (ANI) and digital DNA–DNA hybridization (dDDH) is necessary, following standard taxonomic best practices for the genus (Clarke et al.^ 2022^; Chaidez Oliveira 2024).

Finally, the use of the neighbor-joining phylogenetic method places BPA‑6 on a unique branch with robust bootstrap support (> 70%), consistent with standard practice for bacterial taxonomy; however, we advocate multi-locus sequence analysis (MLSA) using protein-coding genes (e.g. gyrB, atpD, recA, trpB, rpoB) to refine its phylogenetic placement and confirm novelty (Guo et al., 2008; Lapierr, 2012).

### Antimicrobial activity

The agar well-diffusion assay demonstrated that both the culture supernatant and the ethyl acetate extract of *Streptomyces* sp.BPA‑6 inhibited *S. aureus* ATCC6538P, *B. subtilis* ATCC6633, and *C. albicans* ATCC10231, but exhibited no apparent activity against Gram‑negative bacteria (*E. coli, K. pneumoniae and P. aeruginosa*). The inhibition zones recorded for the crude ethyl acetate extract (42.3 ± 3.8 mm, 41.7 ± 3.5 mm, and 48.7 ± 2.1 mm for *S. aureus, B. subtilis, and C. albicans*, respectively) substantially exceeded those observed for the aqueous supernatant (30.3 ± 0.9 mm, 33.3 ± 1.5 mm, and 33.0 ± 1.4 mm), and were also larger than zones produced by gentamycin (29 mm and 36 mm for *S. aureus* and *B. subtilis*, respectively). This pattern aligns with several recent reports where ethyl acetate extracts of *Streptomyces* strains showed markedly stronger inhibitory effects—particularly towards Gram-positive and fungal pathogens—than culture filtrates alone (Bautista-Crescencio et al., 2023; Rammali et al., 2024).

Selective antagonism towards Gram-positive bacteria is common among *Streptomyces*–derived bioactives. These bacteria typically possess thick peptidoglycan layers susceptible to a wide range of extracellular molecules, including polyketides, lipopeptides, and glycopeptides that are effectively extracted by ethyl acetate solvents (Nisha et al., 2025). In contrast, Gram-negative bacteria’s outer membrane frequently restricts penetration of such hydrophobic molecules, explaining the lack of activity observed in this study (Bautista-Crescencio et al., 2023).

The potent inhibition of *C. albicans* by the crude extract—significantly greater than that of the supernatant—is particularly notable. Similar *Streptomyces* ethyl acetate extracts have previously demonstrated fungicidal activity against *Candida glabrata* and resistant strains of *C. albicans*, mediated by metabolites targeting sterol biosynthesis and membrane integrity (Bautista-Crescencio et al., 2023). Although specific secondary metabolites, such as fatty acid esters, terpenoids, and peptide‑like compounds, are widely implicated in both antibacterial and antifungal activity in actinomycetes (Nisha et al., 2025).

Comparatively, *Streptomyces* strain SCJ isolated from Moroccan garden soil produced an ethyl acetate extract that exhibited strong, broad-spectrum activity against both *S. aureus, C. albicans,* and even selected clinical drug-resistant Gram-negative bacteria (zone diameters reaching > 30 mm), indicating that ecological origin and genetic background influence the metabolite spectrum (Rammali et al., 2024). The absence of Gram-negative activity in BPA-6 may therefore reflect a narrower metabolomic profile or differing biosynthetic gene cluster (BGC) expression under ISP-2 culture conditions.

Considering minimal inhibition concentrations, and compared with other *Streptomyces*-derived extracts, the MIC values recorded for BPA-6 demonstrate relatively high activity. For example, the ethyl acetate extract of *Streptomyces achromogenes* TCH4 (from Thai mangrove sediments) exhibited MICs of 125–250 µg/mL against *S. aureus* and *B. subtilis*, with minimum bactericidal concentrations (MBCs) ranging from 500 µg/mL to > 4,000 µg/mL, significantly higher than the 1,000 µg/mL MBC reported for BPA-6 (Tangjitjaroenkun et al., 2021). Meanwhile, antimicrobial extracts of *Streptomyces* strains from Amazonian soil have shown MICs as low as 15.6 µg/mL (*Streptomyces* 3323 EtOAc against *S. pneumoniae*), typically reaching 250 µg/mL against *S. aureus* and *C. albicans* (Oliveira et al., 2024). These results place the BPA-6 extract within the efficacy range described in these studies, particularly against Gram-positive bacteria and yeasts. However, some studies on specialized metabolites show significantly higher efficacy: an ethyl acetate extract of *Streptomyces misakiensis* yielded MICs of 0.5 µg/mL and MICs of 1.5 µg/mL and 1 µg/mL against multidrug-resistant bacteria and fungi, attributed to compounds such as ursolic acid methyl ester (Devi et al., 2023). These exceptionally low MICs highlight the disparity in activity among *Streptomyces* species, suggesting that BPA-6 may produce moderately potent, but nonetheless promising, antimicrobial metabolites.

In general, the MICs of BPA-6 extract are favorable compared to other ethyl acetate extracts of environmental *Streptomyces*, especially considering its efficacy against Gram-positive pathogens and *C. albicans*. Although not among the most extreme, its efficacy combined with the simplicity of the extract (crud extract) and moderate MMC values supports further work to isolate and characterize pure compounds enriched in activity.

### GC–MS analysis

The GC–MS total ion chromatogram (TIC) of the ethyl acetate extract of *Streptomyces* sp. BPA-6 revealed a chemically rich volatilome. A prominent peak at ~ 21–22 min retention time, along with numerous smaller peaks between 10 and 45 min, suggests the presence of over 40 volatile organic compounds (VOCs). This chemical diversity aligns with earlier reports on *Streptomyces* strains isolated from various ecological niches (Kaur et al., 2022; Schulz-Bohm et al., 2017; Liu et al., 2022; Li et al., 2020).

For instance, marine-derived *Streptomyces levis* KS46 was shown to produce over 42 VOCs via GC–MS, including fatty acid esters, hydrocarbons, alcohols, phenols, and terpenoids (Kaur et al., 2022). Likewise, rhizospheric and marine strains, like *Streptomyces* sp. NKM1, produces a spectrum of volatiles such as phthalates, alkane, and sterols (Sholkamy et al., 2023; Singh & Dubey, 2018). These patterns suggest that BPA-6 may share a core actinobacterial volatilome while retaining strain-specific metabolic features.

From a chemometric point of view, terpenoids and isoprenoids are hallmark VOCs in *Streptomyces*, often including geosmin, 2-methylisoborneol, and various sesquiterpenes responsible for the characteristic earthy aroma of soil. These compounds are implicated in spore dispersal through insect attraction (Shepherdson et al., 2023). Surveys of industrial and soil-derived strains consistently identify terpenoids as dominant VOCs (Liu et al., 2022; Saha et al., 2020), suggesting that BPA-6 may similarly produce these, though likely better captured using headspace techniques than solvent extracts. Also, aliphatic hydrocarbons, such as tetradecane and hexadecane, are common in marine and soil-associated *Streptomyces* species (Sholkamy et al., 2023; Singh & Dubey, 2018). These long-chain compounds are often byproducts of fatty acid metabolism and are frequently detected in ethyl acetate extracts. In addition, fatty acid esters and phthalates, particularly methyl and ethyl esters of palmitic, stearic, and linoleic acids, are well-documented actinobacterial VOCs (Kaur et al., 2022; Sholkamy et al., 2023; Singh & Dubey, 2018; Elbendary et al., 2021). *Streptomyces* cheonanensis, for example, activity produces phthalate derivatives like diethyl phthalate and 2-methylbutyl propyl phthalate, both with broad-spectrum antimicrobial (Barakat & Beltagy et al., 2015). Similar phthalates were detected in the BPA-6 extract, possibly explaining its bioactive profile.

Phenolic compounds, including 2,4-di-tert-butylphenol, are frequently found in strains like *Streptomyces* TOR3209 and are associated with antifungal and antioxidant activity (He et al., 2022). Given their recurrence across actinobacteria, they may also be part of BPA-6’s volatile repertoire. Other compounds, including long-chain alcohols (e.g., 1-hexadecanol), ketones, sterols (e.g., γ-sitosterol), and triterpenes (e.g., β-amyrin), have been reported in marine and rhizosphere isolates (Osama et al., 2022; Sholkamy et al., 2023; Singh & Dubey, 2018), and their presence in BPA-6 would be metabolically consistent.

Many VOCs produced by actinobacteria possess notable biological activities—antimicrobial, antifungal, antioxidant, cytotoxic, and anti-inflammatory (Barakat & Beltagy et al., 2015; He et al., 2022; Ngamcharungchit et al., 2023). In particular, phthalates and phenols are well-documented antimicrobial agents, while sterols and triterpenes have demonstrated cytotoxic and anti-inflammatory properties (Zhao et al., 2022).

Ecologically, compounds such as geosmin and 2-methylisoborneol facilitate spore dispersal by attracting invertebrates, enhancing the organism’s ecological fitness (Jones & Elliott, 2017; Shepherdson et al., 2023). Some VOCs, such as those from *Streptomyces* TOR3209, can also modulate plant development and signaling (He et al., 2022). However, the identification of metabolites with known anti-biofilm, antioxidant, anticancer, and cytotoxic activities suggests that *Streptomyces* sp. BPA-6 harbors significant pharmaceutical potential and warrants further purification, structure elucidation, and bioassay-guided fractionation to isolate and characterize the active constituents.

### UHPLC-HRM/MS analysis

The HPLC–MS analysis of the ethyl acetate extract of *Streptomyces* sp. BPA-6 revealed a complex and diverse metabolic profile. However, the detection of antibiotic-related metabolites is consistent with the well-known biosynthetic potential of *Streptomyces*, which is responsible for the production of numerous clinically relevant secondary metabolites. Among over 40 detected compounds, key representatives such as nigericin, erythromycin, and glycosyl sugar derivatives stand out for their known bioactive properties. In addition, smaller molecules like amino acids, sugars (raffinose, galactinol), and minor lipids likely contribute to osmoregulation and carbon storage. These findings support the view that BPA‑6 is a rich source of structurally and functionally distinct secondary metabolites, reflecting the strain’s rich biosynthetic potential. Such chemical breadth mirrors findings in other *Streptomyces* metabolomic surveys; for example, Wang et al. (2022) reported over 120 unique metabolites—including antibiotics, herbicides, and plant growth regulators—produced by *Streptomyces lydicus* M01 using a UPLC-QTOF-MS platform, underscoring the genus’s metabolic versatility. Likewise, untargeted LC-HRMS profiling of ethyl acetate extracts from Nepalese *Streptomyces* isolates annotated dozens of molecular families, from polyketides to sugar alcohols, via GNPS molecular networking (Bhattarai et al., 2022). Together, these studies confirm that high-resolution UHPLC-MS workflows are indispensable for capturing both dominant and trace metabolites in actinobacterial extracts.

Among the detected secondary metabolites, nigericin (m/z 723.4681, RT 11.48 min), erythromycin (m/z 734.4685, RT 11.49 min) and glycosyl sugar derivatives stand out as canonical antibiotics for their known bioactive properties. Similar observations were made by Díaz-Cruz and Bignell (2024), who used untargeted MS/MS and molecular networking to link the presence of nigericin-type polyethers to corresponding non-ribosomal peptide synthase (NRPS) and polyketide synthase (PKS) gene clusters in *Streptomyces* sp. 11-1-2. The co-occurrence of macrolides and ionophores in BPA-6 not only highlights conserved biosynthetic pathways across *Streptomyces* species but also suggests potential for co-production of synergistic antimicrobial agents, as reported in mixed-strain fermentations of related actinobacteria (Wang et al., 2022). In addition, the detection of smaller molecules like amino acids, sugar-related primary metabolites—such as raffinose (m/z 503.1617, RT 1.90 min), β-D-allopyranose (m/z 179.0560, RT 2.80 min), and galactinol (m/z 341.1088, RT 4.00 min)—is in line to the findings reported by Gopalakrishnan et al. (2021), who compared HPLC profiles of five *Streptomyces* strains and similarly reported various oligosaccharides and sugar alcohols implicated in osmoprotection and cell–cell signaling. Additionally, Bhattarai et al., (2022) described sugar phosphates and glycosylated peptides in their ethyl acetate extracts, attributing these to both housekeeping metabolism and possible biofilm-modulating roles. The presence of such compounds in BPA-6 suggests adaptive strategies for environmental stress tolerance and intermicrobial interactions.

The detection of nigericin—a polyether K⁺/H⁺ ionophore with potent antibacterial, antiprotozoal, and toxic activities—aligns with its well-documented occurrence in *Streptomyces* spp. (Dembitsky, 2022) and underscores BPA‑6's potential as a natural reservoir of such clinically relevant antibiotics. Furthermore, the presence of erythromycin, the macrolide first isolated from *Saccharopolyspora erythreus* (related to *Streptomyces*) in the early 1950s, is strong evidence of conserved macrolide biosynthetic pathways within BPA‑6. Coupled with detection of auristatinE—a synthetic derivative of the dolastatin family known for high antimitotic activity—this metabolome suggests an unexpected hybrid of natural and action-oriented molecules, some possibly cryptogenic gene cluster products, as often unearthed in modern *Streptomyces* genome mining efforts (Alam et al. 2022).

Additionally, Erythromycin (RT 11.49 min), a macrolide antibiotic, was confirmed in the extract. As a ribosomal 50S subunit binder, its detection suggests the presence of polyketide biosynthetic gene clusters in BPA-6. Netilmicin (RT 4.181 min), an aminoglycoside antibiotic typically used against Gram-negative bacteria, was also present and highlights the strain’s ability to produce ribosome-targeting metabolites (Robertsen & Musiol-Kroll, 2019).

The analysis also revealed several lipid-related compounds. Tetracosanoic acid (RT 14.23 min), a long-chain saturated fatty acid, and PC(20:5/P-18:1) (RT 8.651 min), a complex phospholipid containing eicosapentaenoic acid (EPA), were identified. These molecules may contribute to membrane integrity, host immune modulation, and cell signaling (Kaczmarek & Boguś, 2021). Although PC(20:5/P-18:1) was not prominently detected in the extract, its identification through spectral matching suggests low-abundance membrane lipid derivatives.

Also, the polyketide Cytosporone C (RT 9.829 min) was detected and is known to act as a nuclear receptor (NR4A1) ligand with potential anticancer properties. The extract also contained N-[3-(1-piperidinomethyl)phenoxy]propylamine (RT 2.245 min), a synthetic amine analog potentially acting on histamine receptors or CNS targets (Szczepańska et al., 2023).

In this study, metabolite profiling by UHPLC–HRMS/MS and GC/MS allowed the annotation of several secondary metabolites, including compounds previously reported to exhibit antimicrobial properties. However, since bioassay-guided fractionation was not performed, the antimicrobial activity observed in the crude extract cannot be unequivocally attributed to individual metabolites. The activity may instead arise from the combined or synergistic effects of multiple compounds. Future work involving chromatographic fractionation and purification will be required to identify the metabolites primarily responsible for the observed bioactivity.

Overall, these findings indicate that *Streptomyces* sp. BPA-6 produces a chemically diverse array of metabolites with known or predicted antimicrobial, anti-inflammatory, cytotoxic, and signaling activities. The presence of structurally diverse compounds such as erythromycin, nigericin, and cytosporone C underscores the strain’s value as a promising source of bioactive natural products.

## Conclusion

This study highlights the remarkable bioactive potential of *Streptomyces* sp. BPA-6, an isolate from Algerian bees-collected pollen. Through a combination of antimicrobial assays, GC–MS, and HPLC–MS analyses, the strain was shown to produce a wide range of volatile and non-volatile secondary metabolites, including potent antibiotics such as erythromycin, nigericin, and netilmicin, as well as polyketides, lipids, and glycosylated compounds. The observed antimicrobial activity, particularly against *S. aureus*, *B. subtilis*, and *Candida albicans* reinforces the pharmaceutical interest of actinobacteria from unexplored habitats such as pollen. Furthermore, the detection of rare or structurally diverse molecules suggests that BPA-6 harbors unique biosynthetic gene clusters. Future work should focus on genome sequencing and annotation to identify the biosynthetic pathways responsible for these metabolites. Coupling genomic data with metabolomics and transcriptomics could enable the activation of cryptic gene clusters and produce novel compounds. In addition, bioassay-guided fractionation and structural elucidation of purified compounds are required to evaluate their pharmacological profiles and therapeutic applications. The integration of synthetic biology tools and heterologous expression systems could also facilitate the large-scale production of selected metabolites. Overall, *Streptomyces* sp. BPA-6 represents a promising candidate for natural product discovery and biotechnological exploitation.

## Supplementary Information

Below is the link to the electronic supplementary material.

Supplementary file1 (DOCX 1084 KB)

## Data Availability

All the data in the article are available from the corresponding author upon reasonable request.
