# Supplementary material for: Integrated GC–MS and UHPLC–HRMS/MS profiling of bioactive compounds from Streptomyces sp. BPA-6 isolated from bees-collected pollen
Source: Metabolomics. 2026 May 24;22(3):83. doi: 10.1007/s11306-026-02427-3 (PMC13199188; doi:10.1007/s11306-026-02427-3)

**Integrated GC–MS and UHPLC–HRMS/MS profiling of bioactive compounds from *Streptomyces* sp. BPA-6 isolated from bees-collected pollen.**

Hani Belhadj^1^, Mohamed Mokhnache^1^, Ahmed Mohamed Bachir Alien^1^, Marika Pellegrini ^2^, Sara Palmieri^3^, Federico Fanti ^3^, Daoud Harzallah^1^, Tarek H. Taha^4^, Walid Elfalleh^4^, Stefania Garzoli^5^*, Hamdi Bendif ^4^*

*^1^Laboratory of Applied Microbiology, Faculty of Natural and Life Sciences, University Ferhat Abbas Setif 1, Setif, 19137, Algeria;* [*hani_belhadj@yahoo.fr*](mailto:hani_belhadj@yahoo.fr)*;* [*mmokhnache@yahoo.fr*](mailto:mmokhnache@yahoo.fr)*;* [*ahmedestudio@gmail.com*](mailto:ahmedestudio@gmail.com)*; harzaldaoud@yahoo.co.uk*

*^2^Department of Life, Health, and Environmental Sciences, University of L'Aquila,67100 L'Aquila, Italy;* [*marika.pellegrini@univaq.it*](mailto:marika.pellegrini@univaq.it)

*^3^Department of Bioscience and Technology for Food, Agriculture and Environment, University of Teramo, via Renato Balzarini 1, 64100 Teramo, Italy;* [*spalmieri@unite.it*](mailto:spalmieri@unite.it)*; ffanti@unite.it*

*^4^ Department of Biology, College of Science, Imam Mohammad Ibn Saud Islamic University (IMSIU), Riyadh 11623, Saudi Arabia;* wbelfallah@imamu.edu.sa*;* thali@imamu.edu.sa; *hlbendif@imamu.edu.sa*

*^5^ Department of Chemistry and Technologies of Drug, Sapienza University, P. le Aldo Moro, 5,00185 Rome, Italy;* [*stefania.garzoli@uniroma1.it*](mailto:stefania.garzoli@uniroma1.it)

****Correspondence:*** [*stefania.garzoli@uniroma1.it*](mailto:stefania.garzoli@uniroma1.it)*;* [*hlbendif@imamu.edu.sa*](mailto:hlbendif@imamu.edu.sa)


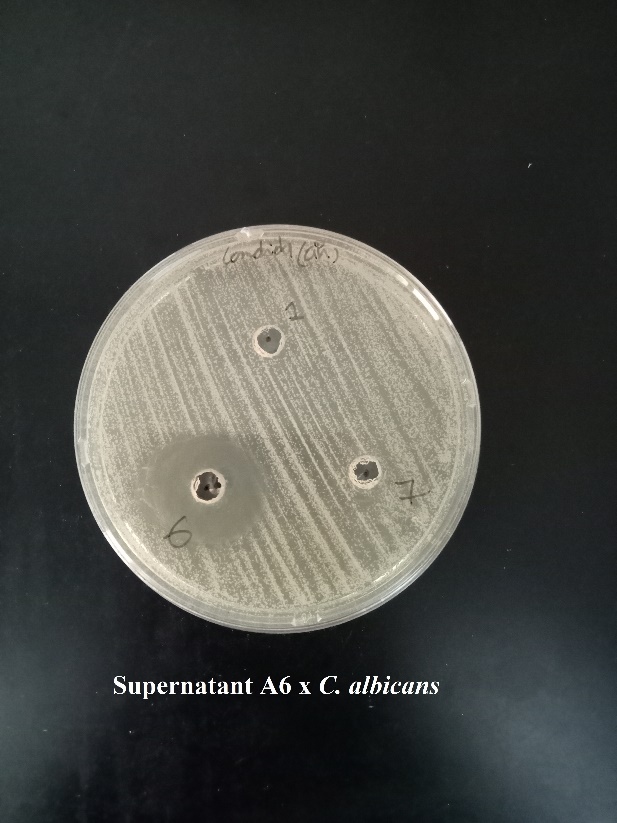

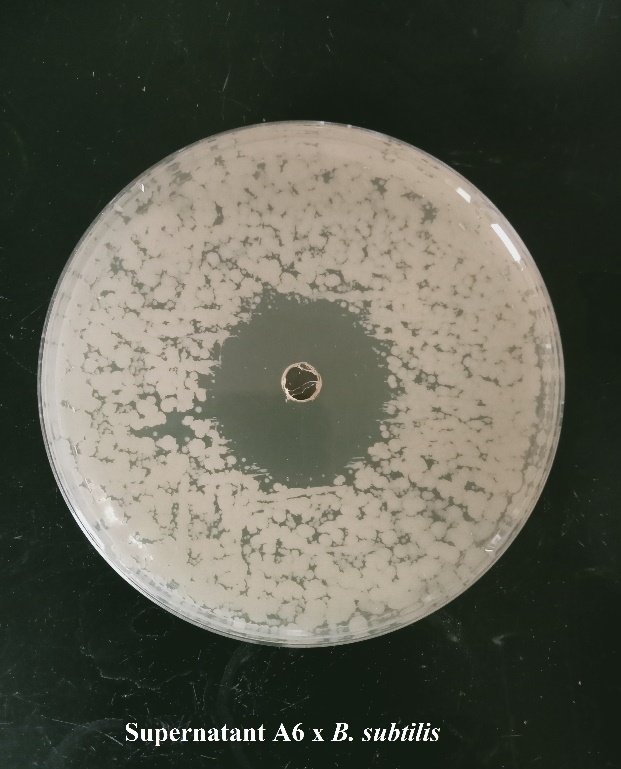

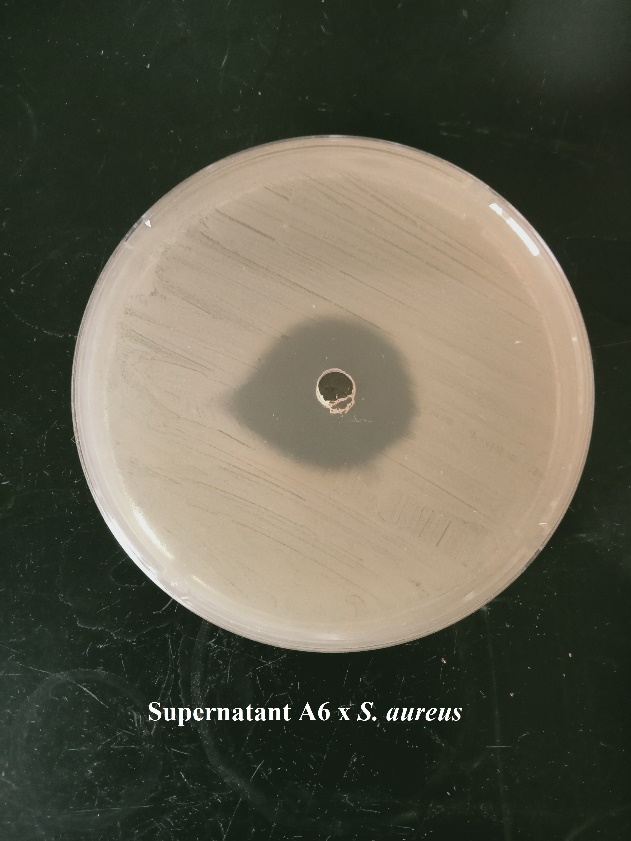

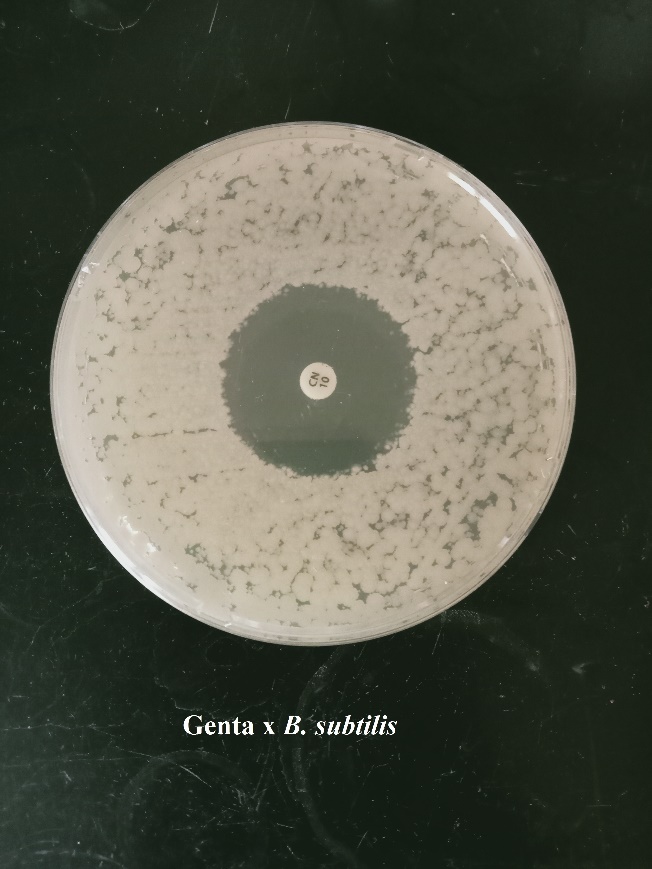

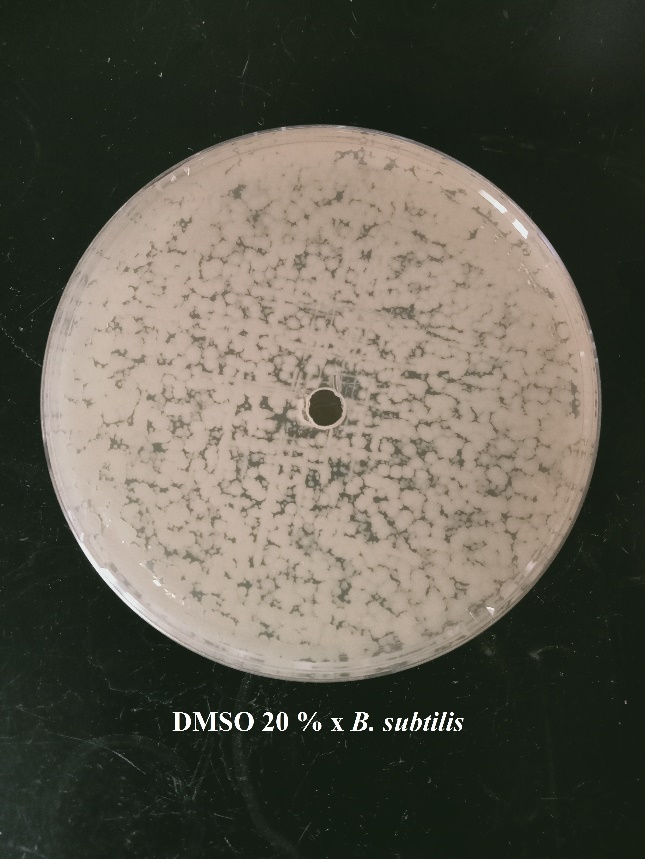

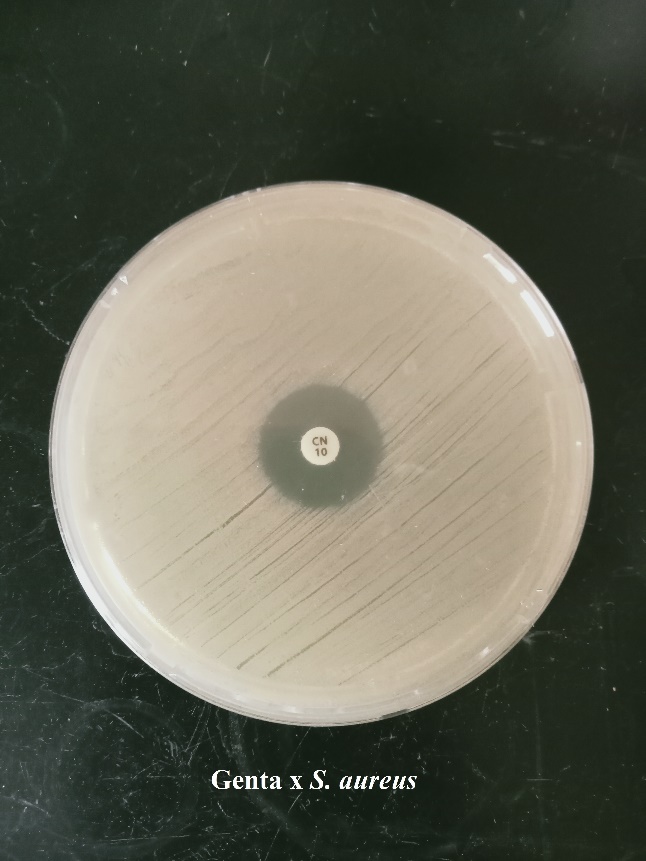

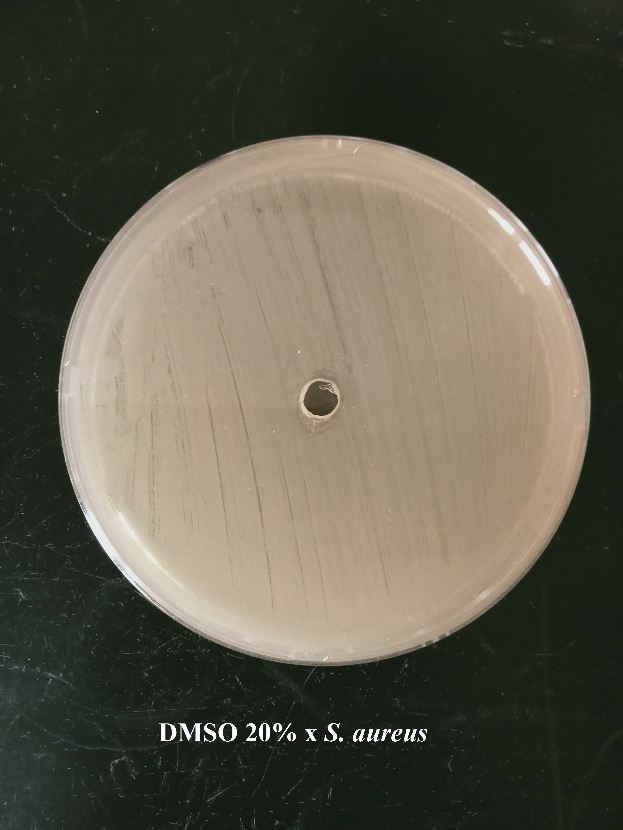

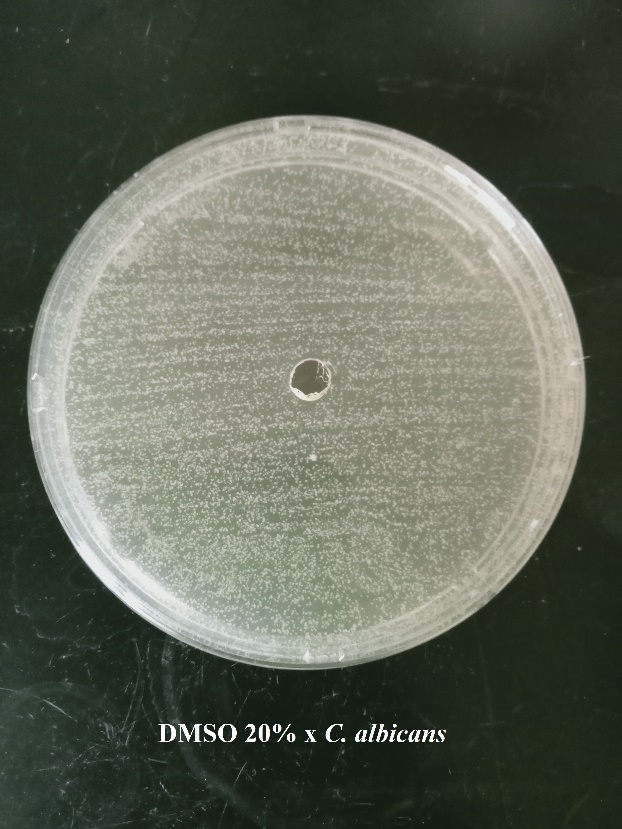

Supplement: Supplementary file 1 — Supplementary file1 (DOCX 1084 KB) [file 11306_2026_2427_MOESM1_ESM.docx]
